# In Vivo Diuretic Activity and Anti-Hypertensive Potential of *Hibiscus sabdariffa* Extract by Inhibition of Angiotensin-Converting Enzyme and Hypertension Precursor Enzymes

**DOI:** 10.3390/foods13040534

**Published:** 2024-02-09

**Authors:** Abdoudramane Sanou, Kiessoun Konaté, Lazare Belemnaba, Hemayoro Sama, Kabakdé Kaboré, Roger Dakuyo, Mathieu Nitiéma, Mamoudou Hama Dicko

**Affiliations:** 1 Laboratory Biochemistry, Biotechnology, Food Technology and Nutrition, Department of Biohemistry and Microbiology, University Joseph KI-ZERBO, Ouagadougou 03 BP 7021, Burkina Faso; 2 Applied Sciences and Technologies Training and Research Unit, Department of Biochemistry and Microbiology, University of Dedougou, Dedougou 09 BP 176, Burkina Faso; 3 Department of Traditional Medicine and Pharmacopoeia and Pharmacy, Institute of Research in Health Sciences/National Centre for Scientific and Technological Research (MEPHATRA PH/IRSS/CNRST), Ouagadougou 03 BP 7034, Burkina Faso

**Keywords:** *Hibiscus sabdariffa*, angiotensin-converting enzyme, diuretic activity, hypertension

## Abstract

Aqueous extracts of calyx from *Hibiscus sabdariffa* (HS) (roselle) are highly appreciated for their nutritional and therapeutic effects, especially as anti-hypertensive substances. This study aimed to evaluate their anti-hypertensive potential through an in vitro inhibition assay of angiotensin-converting enzyme (ACE) and hypertension precursor enzymes and to assess the in vivo diuretic activity of HS. Results showed that HS extract inhibited enzymes belonging to several classes, such as α-amylase, trypsin, chymotrypsin, xanthine oxidase, lipoxygenase, and angiotensin-converting enzyme. In particular, enzymatic kinetics of ACE indicated a competitive inhibition fashion of HS extract. Furthermore, the extracts showed remarkable diuretic and natriuretic effects at doses of 50 mg/kg/bw, 100 mg/kg/b.w, and 200 mg/kg.b.w. These activities can be explained by the high content of phenolic compounds and essential amino acids. Roselle could be a potential source of nutraceuticals and anti-hypertensive bioactive compounds.

## 1. Introduction

Many physiological disturbances are nowadays at the origin of many diseases (hypertension, obesity, dyslipidemia and hyperglycemia) [1]. Among these diseases, the most important risk factor for cardiovascular disease is hypertension, a condition in which the blood vessels have persistently high pressure [2]. In low- and middle-income countries, the prevalence of hypertension has increased in recent years. Indeed, in these countries, the prevalence of hypertension among adults is estimated to be 31.5%, or 1.04 billion people, while high-income countries record a prevalence of 28.5%, or 349 million people [3]. Unfortunately, the genesis of hypertension has been associated with several factors including (i) increased sympathetic nervous system activity, which may be related to increased exposure or response to psychosocial stress; (ii) overproduction of sodium-retentive and vasoconstrictor hormones; (iii) high sodium intake over a long period of time; (iv) inadequate dietary intake of potassium and calcium; and (v) increased or inappropriate renin secretion with consequent increased angiotensin and aldosterone production; deficiencies in vasodilators, such as prostacyclin, nitric oxide, and natriuretic peptides; diabetes mellitus; insulin resistance; obesity; increased activity of vascular growth factors; alterations in adrenergic receptors that influence heart rate, inotropic properties of the heart, and vascular tone; and alterations in cellular ion transport. Research has also considered that endothelial dysfunction, increased oxidative stress, vascular remodeling, and decreased compliance are causes of hypertension and contribute to its pathogenesis [4]. Particularly, according to many studies, 60–70% of hypertension is due to obesity, and obese patients are 3.5 times more likely to develop high blood pressure [5]. Patients with diabetes mellitus have increased peripheral artery resistance associated with hyperinsulinemia and hyperglycemia induced by insulin resistance. Both mechanisms raise systemic blood pressure, resulting in hypertension [6]. Recent studies also reveal that inflammatory mechanisms in certain organs including the kidneys, blood vessels, and brain are precursors to hypertension [7]. In addition to these indirect precursors, the angiotensin-converting enzyme (EC 3.4.15.1, ACE) is a zinc metallopeptidase that cleaves carboxy-terminal dipeptides from several peptides. ACE remains a key enzyme in hypertension occurrence. Indeed, ACE is an enzyme that activates angiotensin I (hypertensive pro-hormone) into an active hormone (angiotensin II), hence its crucial role in bradykinin degradation and vasodilation [8]. In this way, constant blood pressure is maintained by the hypertensive peptide, angiotensin II [9]. Therefore, the inhibition of ACE activity is a candidate treatment for the control of hypertension [10]. Synthetic drugs including ACE inhibitors (e.g., captopril) are already used for the treatment of hypertension, but are unfortunately associated with side effects (hypotension, cough, taste disorders, and angioedema) [11]. In addition, the cost of treatment with modern medicine is very high and beyond the reach of the majority of African populations, who generally resort to medicinal plants for their health. Thus, research interest in the development of anti-hypertensive drugs has increased considerably in recent years, and various medicinal plants have been studied. In this respect, HS has an excellent therapeutic profile, as it is rich in bioactive molecules such as organic acids (citric acid and hydroxycitric acid lactone), phenolic compounds (protocatechuic acid: polyphenols; flavonoid derivatives: gossypetin-3-glucoside, gossypetin-8-glucoside; and anthocyanins: hibiscin, cyanidin-3-β-D-glucoside, delphinidin, sabdaretin, etc.), and amino acids [12,13]. Additionally, its anti-hypertensive therapeutic property is mainly related to its diuretic and angiotensin-converting enzyme (ACE) inhibitory power, but also to its antioxidant, anti-diabetic, and anti-inflammatory activities. The aim of this study is to evaluate the antihypertensive potential of HS extract through the in vitro inhibition assay of angiotensin-converting enzyme (ACE) and hypertension precursor enzymes, as well as to assess its in vivo diuretic activity.

## 2. Materials and Methods

### 2.1. Plant Material

The calyxes of *Hibiscus sabdariffa* (roselle) were obtained in Bobo-Dioulasso (Burkina Faso) in 2020. The samples were transported to the laboratory, then dried in an electric dryer (Dreyer 1100 VISMARA) for 2 weeks at 37 °C and finely ground (≈0.5 mm size) for the various extractions. A specimen of reference 18015/6975/2020/SA was obtained from the herbarium of the Joseph Ki-Zerbo University, Burkina Faso, by the services of Dr Mohamed CISSE.

### 2.2. Chemicals and Reagents

α-amylase [EC 3.2.1.1; 4-α-D-glucan 4-glucanohydrolase], xanthine oxidase [EC 1.17.3.2, xanthine:oxygen oxidoreductase], lipoxygenase [EC 1.13.11.34, arachidonate: oxygen 5-oxidoreductase], chymotrypsin [EC 3.4.21.1], trypsin [EC 3.4.21.4], N-α-Benzoyl-DL-Arginine p-Nitroanilide (BAPNA), N-Glutaryl-L-Phenylalanine p-Nitroanilide (GPNA), furosemide, Aldactone (containing spironolactone as active compound), linoleic acid, iodine reagent, sodium phosphate, Tween 20, the amino acid kit containing standard amino-acids (≥99%), and cystine were purchased from Sigma-Aldrich.

### 2.3. Extraction

Optimal extraction of phenolic compounds was performed by dissolving roselle in distilled water at a concentration of 40 mg/mL and then subjecting it to intense sonication at 50 °C for 1 h. The reaction mixture was cooled, filtered, freeze-dried, and then stored at room temperature for analyses. For protease inhibitors, extraction consisted of dissolving 100 g of extract in 10 mL of NaCl (0.1 M) for 5 h at room temperature. The supernatant was collected after centrifugation at 150 rpm for 30 min at 4 °C and used for the evaluation of trypsin- and chymotrypsin-inhibitory activities.

### 2.4. Evaluation of the Inhibitory Activity of α-Amylase

The kinetics of α-amylase inhibition by HS extract was studied as previously described [14]. The reaction medium contained 50 μL of phosphate buffer (50 mM, pH = 6.8), 10 μL of α-amylase (10 IU/mL), and 20 μL of varying concentrations of extracts (100; 90; 80, 70, 60, 50, 40, 30, 20, 10, and 1 µg/mL), and was then preincubated at 37 °C for 10 min. Then, 20 μL of soluble starch (5 mg/mL) was added as substrate, followed by further incubation at 37 °C for 15 min. The reaction was stopped by the addition of 20 μL of 1 N HCl, followed by the addition of 100 μL of iodine reagent (5 mM I2 and 5 mM KI). The absorbance was read at 620 nm using a spectrophotometer. The inhibitory activity of α--amylase is determined by Equation (1):

(1) $$\%\;I=\frac{\left(A_{c}-A_{e}\right)*100}{A_{c}}$$

where % I: inhibition percentage; A_C_: absorbance of the negative control without extract (inhibitor), A_e_: absorbance in the presence of the extract (inhibitor).

### 2.5. Evaluation of the Inhibition of Xanthine Oxidase (XO)

Xanthine oxidase (XO) is a metalloenzyme containing molybdenum as cofactor. The inhibition of xanthine oxidase activity by the extracts was determined as previously described [15]. Beforehand, xanthine substrate (0.1 mM) and xanthine oxidase (0.28 U) were prepared in phosphate buffer (50 mM, pH 7.4) and then mixed and directly used. The HS extracts (10 μL) at a concentration ranging from 1 to 100 mg/mL were mixed with 150 μL of sodium phosphate buffer (50 mM, pH 7.5) and 10 μL of a xanthine oxidase solution. The mixture was incubated 25 °C for 10 min. The reaction was then initiated by the addition of the xanthine solution in the medium. The reaction was followed by measuring the absorbance of the reaction medium at 295 nm for 10 min at 25 °C using a spectrophotometer (EPOCH, BioTek Instruments Inc., Winooski, VT, USA). The proportion of inhibition of xanthine oxidase was calculated using the following equation:

(2) $$\%\;I=\frac{\left(V_{0}-V_{e}\right)\times 100}{V_{o}}$$

where I%: percentage of XO inhibition; V_0_: variation of absorbance per min of the test without the extract; V_e_: variation of absorbance per min of the test with the extract

### 2.6. Evaluation of the Inhibitory Activity of Lipoxygenase

Lipoxygenase is a non-heme iron dioxygenases that acts by the removal of a hydrogen H- and the attachment of an oxygen molecule. The principle of this assay is to inhibit the in vitro ability of lipoxygenase to produce leukotrienes and lipoxin in the presence of HS extract. The assay was performed by mixing 2 µL of substrate (1.25 mM linoleic acid) with 2 µL Tween 20, 65 µL NaOH (0.1 N), and distilled water to reach a total volume of 5.14 mL. The mixture was left at 22 °C for 12 h. The enzyme solution (10,000 U/mL) was obtained by preparing type I-B lipoxygenase in borate buffer (200 mM, pH 9) to obtain a final concentration of 820.51 U/mL in the reaction medium. The reaction medium consisted of 3.75 μL of extract (100 µg/mL), 153.75 μL borate buffer (200 mM, pH 9), and 146.25 μL A5-LOX solution. The whole set was homogenized and incubated for 5 min at 25 °C, and then the linoleic acid solution was added (150 μL) [16]. The absorbances of the reaction medium were read at 234 nm after 3 min of reaction at 25 °C. The percentage of A5-LOX inhibition was determined by comparing the reaction rates of the extract to the control using Equation (1).

### 2.7. Determination of the Inhibition of Trypsin and Chymotrypsin

The inhibition of trypsin by HS extract was determined according to Sombié et al. [17]. Thus, 100 µL of trypsin (0.0125 mg/mL) was added to 100 µL of protein extract supernatant and incubated for 5 min; then, 50 µL of N-α-Benzoyl-DL-Arginine p-Nitroanilide (BAPNA) at the concentration 0.8 mg·mL^−1^ was added. The release of p-nitroanilide was monitored at 410 nm using a 96-well spectrophotometer for 25 min.

The chymotrypsin inhibitory activity was determined using the procedure described by Sombié et al. [17]. Approximately 100 μL of the supernatant was added to 100 μL of chymotrypsin (from bovine pancreas, Sigma) at the concentration of 100 μg/mL. The mixture was incubated for 5 min and then 50 μL of substrate N-Glutaryl-L-Phenylalanine p-Nitroanilide (GPNA) at the concentration of 3.2 mg·mL^−1^ was added to the mixture. The release of p-Nitroanilide was monitored for 25 min at 410 nm against a control using a 96-well spectrophotometer. The percentage inhibition of trypsin and chymotrypsin was calculated by the following Equation (1).

### 2.8. Determination of the Inhibition of Angiotensin-Converting Enzyme

The assessment of the inhibition of ACE in vitro was performed by quantifying the hydrolysis of N-(3-[2-furyl]acryloyl)-phenylalanyl glycyl glycine (FAPGG) by ACE [18]. Briefly, FAPGG and samples were individually dissolved in 50 mM Tris-HCl buffer, pH 7.5 containing 0.3 M NaCl. An aliquot of 10 µL of ACE (final reaction activity 25 mU) was added to each well containing 170 µL of FAPPG (0.5 mM) and 20 µL of samples at 37 °C. The buffer was used as a blank (uninhibited reaction), while the ACE-inhibiting drug captopril was used as a positive control and assayed using a similar protocol. Absorbance was read using a spectrophotometer at 345 nm at 5 min intervals for 30 min to determine the reaction rate. The slope of the blank or sample reactions was used to calculate the percentage of ACE inhibition (Equation (2)).

For the determination of the hydrolysis kinetics of FAPGG in the presence of the inhibitors, solutions of growing concentrations of FAPGG (0.05, 0.1, 0.2, 0.4, 0.6, 0.8, and 1 mM) were used. Enzyme activity was calculated by quantifying the decrease in FAPGG concentration by recording the decrease in absorbance at 345 nm. Fitting the curves to the Michaëlis–Menten equation, the kinetic parameters Vmax (maximum speed) and Km (Michaëlis constant) were determined by Equation (3).

(3) $$V_{o}=\frac{\mathrm{Vmax}\;\left[s\right]}{K_{m}+\left[s\right]}$$

The inhibitory constants were calculated from the double reciprocal graph, using Equation (4):

(4) $$m_{i}=m\left(1+\frac{\left[I\right]}{K_{I}}\right)$$

where m_i_ = slope of the line graph in the presence of inhibitor, m = slope of the line graph without inhibitor, [I] = concentration of the inhibitor, and K_i_ = dissociation constant of the inhibitor.

### 2.9. Evaluation of Diuretic Activity

The experimental protocol was approved by the ethical committee of the Institute of Health Sciences Research and a number has been assigned to it: CE-IRSS/2021-08. Seven-week-old Wistar rats (about 150 g) from the laboratory of animal physiology of the University Joseph KI-Zerbo, Burkina Faso, were used for this experiment [19]. All animals were pre-administered with 25 mL/kg/bw of 0.9% NaCl solution, and those with a baseline urinary diuresis of 2 mL were retained for further experimentation. Six groups of mixed male and female rats (four per group) were fasted and given the various treatments orally through a tube:

- Group 1: negative control, the animals received 0.9% NaCl solution;
- Groups 2, 3, and 4: the test batches were treated with an aqueous extract of HS at doses of 50, 100, and 200 mg/kg/bw (HS 50, HS 100, and HS 200), respectively;
- Group 5: hypokalemic positive control where the animals received a furosemide solution at a dose of 10 mg/kg/bw;
- Group 6: hyperkalemic positive control where animals received Aldactone solution at a dose of 25 mg/kg/bw.

The diuretic activity was determined according to the methods used by Kau et al. [20] with slight modifications [19]. Falcon tubes (15 mL) were attached to the experimental device to collect urine and its volume was measured 6, 12, and 24 h after treatments. Urine volumetric excretion was determined according to the following Equation (5):

(5) $$\mathrm{UVE}=\frac{\mathrm{VUE}}{\mathrm{VFO}}\times 100$$

where UVE: urinary volumetric excretion; VUE: volume of urine excreted (expressed as mL.100/g); VFO: volume of fluid overloaded (25 mL·kg^−1^).

After pH measurement, the urine was stored at −20 °C, and electrolytes (sodium and potassium ions) were quantified. The ratio of urinary excretion in the test group to urinary excretion in the control group was used as a measure of the diuretic index of a given dose, and urinary volumetric excretion was used to assess diuretic activity.

### 2.10. Amino Acid Profile

The amino acid profile was obtained by HPLC using the PICO-TAG method described by Bidlingmeyer et al. [21]. Roselle powder (400 mg) was hydrolyzed in acid medium (15 mL 6 N HCl) under an electric oven at 110 °C for 24 h. Following the hydrolysis, the samples were transferred to 50 mL volumetric flasks and the volume was adjusted with milli-Q water. The mixture was evenly homogenized and then filtered (0.45 µm). An aliquot of 10 µL was dried in a vial for 10–15 min under vacuum (65 mTorr) using the PICO-TAG Workstation. Specifically, the drying solution consists of ethanol, water, and trimethylamine (2:2:1). The dried sample is then derivatized with a phenylisothiocyanate (PITC) solution to produce the stable phenylthiocarbamyl (PTC) amino acids. To the derivatized sample, 200 µL of PICO-TAG dilution solution (0.38 µg/µL) was added and then injected to HPLC system. The separation system includes: an injector, a pump, a column, and an oven and is equipped with a UV detector and a control station driven by a computer. The amino acid derivatives were finally separated by elution through a PICO-TAG precolumn (Nova-Pak C18 Guard Column, 60 Å, 4 μm, 3.9 mm × 20 mm) and column (Waters PICO-TAG C18 Column (3.9 × 150 mm)) and then quantified by a UV detector (1 picomole sensitivity) at 254 nm [19]. The content of each of the amino acids (Taax) in the sample is calculated and expressed as a percentage of the dry matter mass according to the equation:

(6) $${\mathrm{Taa}}_{x\;}\left(\%\right)=\frac{\mathrm{Caax}}{\mathrm{Cie}}\times 100$$

where Taax = amino acid content x in % m/m relative to DM; Caax = amino acid concentration x; Cie: initial sample concentration.

### 2.11. Statistical Analysis

All experiments were performed at least in triplicate. Data were averaged mean ± standard error of the mean (SEM, n = 3) and subjected to statistic Tukey analyses to appreciate significant differences (*p* ≤ 0.05 and *p* ≤ 0.01) using XLSAT version 2016. GraphPad Prism version 8.4.3 was used to design the graphs.

## 3. Results

### 3.1. Inhibition of Hypertension Precursor Enzymes

The aqueous extract of HS inhibited the activity of tested enzymes (Table 1). The analysis showed that the inhibitory concentration of the HS extract to reach 50% of inhibition (IC50) for α-amylase, trypsin, chymotrypsin, xanthine oxidase, and angiotensin-converting enzyme activity were 87.125 ± 12.94 µg/mL; 568.762 ± 43.36 µg/mL; 463.77 ± 27.54 µg/mL; 362.5 ± 15.72 µg/mL; and 174.62 ± 9.42 µg/mL, respectively, while a concentration of 100 µg/mL resulted in inhibition in 14.5% of lipoxygenase. In addition, the angiotensin-converting enzyme synthetic inhibitor (captopril) had an IC50 inhibitory activity of 94.56 µg·mL^−1^.

### 3.2. Enzymatic Kinetics of ACE

**Figure 1:**
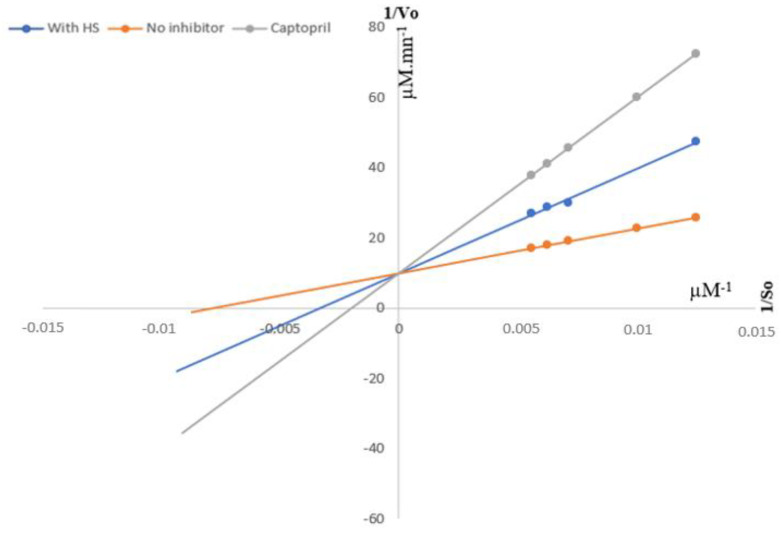
Lineweaver-Burk plots for inhibition of ACE activity in the presence of HS, in the presence of captopril and without inhibitor.

Lineweaver–Burk double inverse plots of ACE enzyme allowed us to determine the type of inhibition in the presence of captopril and aqueous HS extract, compared to control. The extracts did not change the Vmax (6.04 µM·min^−1^), but affected the apparent Km as a function of inhibitor concentration (Table 2). The curves indicated that both HS extract and captopril behaved as competitive inhibitors of ACE (Figure 1).

### 3.3. Diuretic Activity

#### 3.3.1. Effects of Oral Administration of HS Extracts on Weight Loss, pH, and Electrolyte Excretion

**Figure 2:**
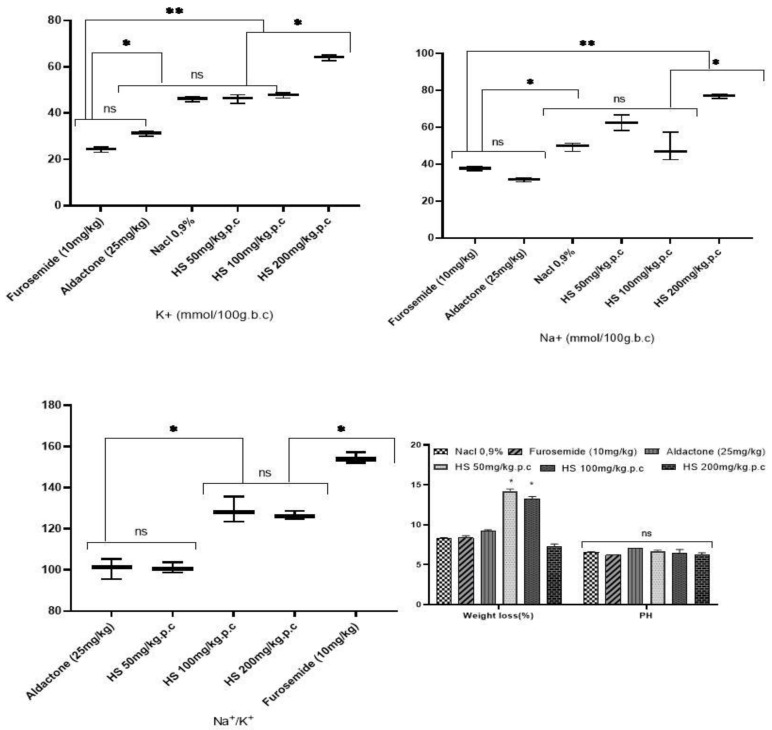
Effect of HS extracts of standard diuretics on the saluretic index, natriuretic activity, urinary pH, and weight dehydration. ns: insignificant difference; (*) significantly different at *p*-value (*p* ≤ 0.5) and (**) and significantly different at *p*-value (*p* ≤ 0.01).

For saluretic index and natriuretic activity, the concentration of 200 mg/kg^−^/b.w showed a significant difference in interest compared to standard diuretics (*p* < 0.001) and extracts of concentrations of 50 mg/kg.b.w and 100 mg/kg.b.w, respectively (Figure 2). The Na^+^/K^+^ ratio showed an interesting effect for 100 mg/kg.b.w and 200 mg/kg.b.w concentrations, which were highly significant compared to Aldactone and slightly weak compared to Furosemide. The pH of urine was similar in the different treatment groups, and the ratio of pre-treatment weight to post-treatment weight showed no significant difference in the controls (positive and negative) and 200 mg/kg.b.w. extract, whereas 50 mg/kg.b.w and 100 mg/kg.b.w. concentrations showed an interesting weight gain.

#### 3.3.2. Effect of Oral Administration of HS Extracts on Diuretic Index and Diuretic Activity

Different treatments induced significant levels of effects on urine urinary volumetric excretion and the diuretic index (Table 3). The fractions of the extracts (50 mg/kg.b.w, 100 mg/kg.b.w, and 200 mg/kg.b.w) showed interesting diuretic activities similar to the positive controls (Furosemide and Aldactone). In addition, the urine output was influenced in a dose-dependent manner by the aqueous administration of our extracts, i.e., the high concentrations presented the high urine outputs.

### 3.4. Amino Acids Profile

**Figure 3:**
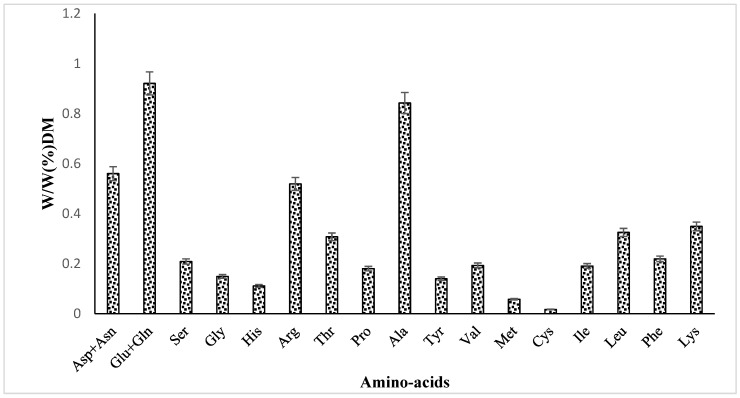
Amino acid profile.

HS extract contained all essential amino acids: methionine, leucine, valine, lysine, isoleucine, phenylalanine, and threonine, except for tryptophan, which could not be quantified (Figure 3). A rank of amino acids according to their content is as follows: Glu + Gln > Ala > Asp + Asn > Arg > Lys > Leu > Thr > Phe > Ser > Val > Ile > Pro > Gly > Tyr > His > Met > Cys.

## 4. Discussion

Hypertension is the major risk factor in the occurrence of cardiovascular disability [22]. An estimated 1.13 billion people suffer from it worldwide [23]. However, its pharmacological management is not sufficiently mastered and involves the use of calcium channel blockers, β-blockers, angiotensin-converting enzyme (ACE) inhibitors, angiotensin II receptor blockers, vasodilators, α-blockers, and diuretics. The approach, namely the use of diuretics as hypotensive agents, is explained by the ability of these drugs to inhibit the Na^+^; K^+^; Cl^−^ co-transporter (NKCC2) on the apical side of Henle’s loop epithelial cells by binding reversibly to its Cl^−^ site, resulting a decrease in the reabsorption of sodium, potassium, and chloride ions, thus leading to natriuresis [24]. The hypotensive effects of diuretics may also be explained by vasodilator activity via the inhibition of tubulo-glomerular feedback and by cyclooxygenase-2-mediated prostaglandin pathways, which may affect renin expression, renal perfusion, and glomerular filtration [25]. On the other hand, ACE is a two-domain (N- and C-terminal) dipeptidyl-carboxypeptidase that is directly involved in the regulation of blood pressure by hydrolyzing angiotensin I to produce angiotensin II. ACE inhibition is a preventive alternative to the occurrence of hypertension via vasoconstriction [26]. Therefore, the search for a drug with bilateral properties (ACE inhibitor and diuretic) is still a challenge. The present study reports the pharmacological characterization of the diuretic and natriuretic effects of HS extracts. Firstly, the analysis showed a dose-dependent influence of HS extracts on urine flow rate compared to standard diuretics, thus showing the importance of the diuretic activity. Similar results were obtained with extracts of *Corrigiola telephiifolia* [22]. In addition, flavonoid-rich extracts are well-known diuretic agents with the ability to modulate various physiological processes associated with diuresis [27]. The stability of the urinary pH indicated that the extracts do not have a negative toxicological influence on the internal environment. The urinary ion profile of the different treatments showed that HS extract at the concentration of 200 mg/kg.b.w exhibited naturalistic activity and saluretic index compared to the standard diuretics. In addition, the 100 mg/kg.b.w and 200 mg/kg.b.w doses showed the best Na^+^/K^+^ ionic ratios. Moreover, the aqueous extract of calyxes increases the excretion of sodium ions over potassium, which is an essential quality of a good diuretic [28]. Then, the second approach showed that the aqueous extract consequently inhibited ACE. Enzymatic kinetics confirmed that aqueous extract of HS, as well as captopril, competitively inhibited ACE. Similar results were found by Ojeda et al. [16], showing comparative inhibitory activity to lisonopril [18]. However, this observed ACE inhibitory activity is attributed to flavonoids and peptides, according to the literature. For flavonoids, the structure of anthocyanins explained their ability to chelate metals with hydroxyl groups, and their flat structure favors the inhibition of metallopeptidases [29]. In particular, the inhibition of ACE by the major anthocyanins of HS was confirmed [18]. Peptides containing tryptophan, proline, or phenylalanine at the C-terminus or branched-chain aliphatic compounds at the N-terminus have good ACE inhibitory activity [30]. In addition, acidic amino acids (Asp and Glu) can chelate zinc atoms, which are cofactors of ACE [31]. This study confirms the hypotensive and anti-hypertensive effects of the Bowman–Birk protease inhibitor, particularly inhibiting trypsin and chymotrypsin [32]. This property contributes to the bioavailability of peptides with ACE inhibitory activity [33]. By inhibiting XO, HS extract may be useful in managing hyperuricemia, an index of gout, and preventing downstream events, including increased reactive oxygen species production, activation of the renin/angiotensin pathway, and inactivation of bradykinin, which links hyperuricemia to hypertension [34]. By inhibiting 5-LOX, HS extract is part of a preventive approach to inflammation and hypertension. Several previous studies have confirmed the ability of HS to inhibit XO [35] and 5-LOX [36]. Indeed, these inhibitory activities of xanthine oxidase and 5-Lipoxygenase indicate that our extracts would inhibit the biosynthesis of potential pro-inflammatory agents including uric acid and leukotrienes LTA4, LTB4, LTC4, LTD4, and LTE4 [37]. Finally, a last approach to reduce postprandial hyperglycemia is the inhibition of carbohydrate-hydrolyzing enzymes in the digestive system. This test was performed by inhibiting α-amylase with HS extract, and corroborated similar studies [1]. The anti-diabetic effect of HS studied is correlated with its biochemical composition, including organic acids, anthocyanins, flavonoids, and polyphenols. These anti-diabetic effects involve the inhibition of pancreatic α-amylase activity, intestinal α-glucosidase, a reduction in the increase in Advanced Glycation End (AGE) in plasma, and an antioxidant effect that may suppress hyperglycemia-induced oxidative stress. Some previous reports have also suggested the benefits of HS for diabetic complications. Indeed, HS reduces triglycerides, cholesterol, and LDL-cholesterol while increasing HDL-cholesterol in diabetic patients. HS consumption decreased systolic blood pressure in type 2 diabetic patients with mild hypertension [25]. This study shows that HS is an important inhibitor of direct and indirect precursor enzymes of hypertension.

## 5. Conclusions

The present study showed that aqueous HS extract was able to inhibit ACE enzyme activity in vitro and showed interesting diuretic and natriuretic effects at different doses. In addition, HS extract was found to be an effective inhibitor of α-amylase, trypsin, chymotrypsin, xanthine oxidase, and lipoxygenase, suggesting a strong potential to limit indirect precursor enzymes of hypertension. Overall, the extracts produced the most promising inhibitory effects on the targeted enzymes in this study. HS calyxes are, therefore, a good candidate for nutraceutical use, not only to cure but also to prevent hypertension by inhibiting blood pressure-regulating enzymes such as ACE.

## Figures and Tables

**Table 1:** Inhibitory concentration (IC50 or %I) of enzymes by HS extract.

Inhibition	IC50 (µg/mL)	% Inhibition
α-amylase	87.125 ± 12.94	-
Trypsin	568.762 ± 43.36	-
Chymotrypsin	463.77 ± 27.54	-
Xanthine oxidase	362.5 ± 15.72	-
Lipoxygenase (100 µg·mL^−1^)	-	14.5 ± 6.52
ACE	174.62 ± 9.42	-
Captopril	94.56 ± 1.31	

**Table 2:** Kinetic parameters of the ACE inhibitor activity of the extract, fraction HS.

Compounds	Vmax (µM.min^−1^)	Km (µM)
No inhibitor	6.04	126.58
With HS	6.04	285.71
With captopril	6.04	500

Vmax: Maximum velocity and Km: Michaelis constant.

**Table 3:** Effect of different treatments on diuretic index and diuretic activity.

Groups	Urinary Volumetric Excretion (%)			Diuretic Index for 24 h	
	6 h	12 h	24 h	DI	Interpretation
Nacl 0.9% (Control)	19.29 ± 3.29	96.45 ± 48.14	173.61 ± 81.57	1	No activity
Furosemide (10 mg/kg)	100 ± 68.43	154.32 ± 100.56	262.34 ± 133.69	1.51 ± 0.77	Important diuretic
Aldactone (25 mg/kg)	87.38 ± 82.47	159.57 ± 105.49	224.07 ± 150.48	1.29 ± 0.86	Important diuretic
HS 50 mg/kg.p.c	55.73 ± 41.79	123.40 ± 78.06	187.10 ± 37.76	1.07 ± 0.21	Important diuretic
HS 100 mg/kg.p.c	27.25 ± 7.1	70.09 ± 50.84	175.23 ± 71.08	1.00 ± 0.48	Important diuretic
HS 200 mg/kg.p.c	31.54 ± 38.63	134.06 ± 71.15	208.99 ± 77.98	1.20 ± 0.44	Important diuretic

DI: diuretic index, UVE: urinary volumetric excretion.

## Data Availability

All data used for this study are included in the study to support our conclusions.
